# Pediatric Sickle Cell Disease in Sudan: Complications and Management

**DOI:** 10.1155/2022/3058012

**Published:** 2022-02-14

**Authors:** Meysaa Talha, Bashier Osman, Safa Abdalla, Hind Mirghani, Iman Abdoon

**Affiliations:** ^1^Clinical Pharmacy Program, Faculty of Pharmacy, University of Khartoum, Khartoum, Sudan; ^2^Department of Pharmacology, Faculty of Pharmacy, University of Khartoum, Khartoum, Sudan; ^3^Consultant Pediatrician and Hematologist, Gaafar Ibnauf Pediatric Tertiary Hospital, Khartoum, Sudan

## Abstract

**Background:**

Sickle cell disease (SCD) is a life-threatening genetic disorder due to the formation of sickle hemoglobin molecule (HbS) that polymerizes in hypoxic conditions leading to SCD-related complications. Different approaches have been used in the management of SCD including symptomatic management, supportive management, and preventive management.

**Objectives:**

To assess the management of SCD in pediatric patients in Gaafar Ibnauf Referral Hospital in Khartoum locality, Sudan.

**Method:**

A descriptive, retrospective, hospital-based study was conducted in Gaafar Ibnauf Hospital using a data collection sheet. The study included all medical files of pediatric patients with SCD attending the hospital during the period from the first of April 2018 to the first of July 2018. The data were analyzed using descriptive statistics and the chi-square test. *P* < 0.05 was considered statistically significant.

**Results:**

Out of 207 pediatric patients, 53.1% were females (mean age of 7.5 ± 3.1 years), with a 1.1 : 1 female:male ratio and low socioeconomic status. Only 4.3% of participants had health insurance. The Messeryia tribe in western Sudan had the highest prevalence of the disease among the Sudanese tribes (11.1%). Vaso-occlusive crisis (33.3%), infections (13.5%), and neurological complications (10.6%) were the most frequent complications reported during routine visits. After initiation of management, only 3.4% of pediatric patients had hemolytic crises, and 1.4% of the anemic patients had splenomegaly. 100% of patients received folic acid, 73.9% used hydroxyurea, and 69.6% underwent blood transfusion for the management of SCD. Prophylactic penicillin was prescribed for 15% of patients, and 41.1% were immunized with pneumococcal vaccine (PPSV23). Most patients had been scheduled for planned follow-up visits every 3–6 months (93.2%). Hydroxyurea and blood transfusion significantly reduced fever and vaso-occlusive crisis.

**Conclusion:**

The SCD treatment protocol in Gaafar Ibnauf Children's Hospital, involving preventive and symptomatic therapy, is consistent with the internationally implemented protocols for SCD management. However, immunization and prophylactic penicillin approaches are deficient.

## 1. Introduction

Sickle cell disease (SCD) is a life-threatening genetic disorder due to the formation of sickle hemoglobin molecule (HbS) with low affinity to oxygen. In the presence of hypoxia, HbS polymerizes and ultimately results in sickled red blood cells that rupture leading to hemolytic anemia. Moreover, the sickled cell also results in vascular occlusion causing tissue infarction, organ damage, and pain [1].

SCD affects many people throughout the globe, particularly those descending from Sub-Saharan Africa, the Middle East, and South Asia. In general, SCD exists in the malarial regions of tropical areas. However, migration from a malarial area increases the number of children with SCD in Europe and North America [2–4]. In the United States, SCD affects 1 in 500 African Americans. It is estimated that the population of sickle cell disease is approximately 4.4 million people, whereas 43 million are estimated to have sickle cell trait [5]. In Sudan, sickle cell disease is a major health problem in certain parts of the country, particularly the western region. The HbS allele frequently exists among the Misseriya tribe, and it is estimated to range from 18.2% in Kordofan to 30.4% in Darfur [6, 7].

The main complications of SCD include pain syndromes, hemolytic anemia, and organ damage/failure [8]. The incidence of complications varies with age from infancy through adult life. Recurrent pain episodes, the hallmark of the disease, result from acute painful vaso-occlusive crises (VOCs) when the sickled cells block the blood vessels. The pain is usually nociceptive in nature, which varies from patient to patient. It could be acute or chronic, somatic or visceral, unilateral or bilateral, localized or diffuse. Generally, pain affects long bones, joints, and the back; however, the scalp, face, jaw, abdomen, and pelvis may be involved [8]. In addition, bacterial infections and anemia are common complications in children with SCD [9,10]. Acute chest syndrome (ACS), splenic sequestration, and multiorgan damage are considered life-threatening conditions that require immediate hospitalization. Cardiopulmonary complications, including cardiomyopathy, pulmonary hypertension, and sudden cardiac death, are the most common causes of morbidity and mortality. Children with SCD are at high risk for developing thrombosis and stroke. SCD is now recognized as a systemic disease, which causes widespread tissue/organ injury, including inflammatory dysfunction and coagulation abnormalities. All children with SCD should be screened annually with transcranial Doppler ultrasonography (TCD). It is highly recommended for children with SCD from 2–16 years of age [8, 11–13].

The management of SCD and its complications requires major approaches, including supportive management, symptomatic treatment, as well as preventative measures. The ultimate goal of treatment is to alleviate the symptoms and to maintain a good quality of life [8, 14].

A published guideline [15] strongly recommends daily oral prophylactic penicillin for children with SCD up to the age of 5 years to reduce the risk of infections. In addition, immunization especially pneumococcal vaccination, was strongly recommended by SCD treatment guidelines [15,16]. The American Society of Hematology (ASH) guideline recommends opioids for the treatment of acute pain associated with vaso-occlusive crisis. For adults who have SCD-related chronic pain, the ASH guideline suggests the use of nonopioid analgesics such as NSAIDs, duloxetine, gabapentinoids, or tricyclic antidepressants, as options for pain management [16]. Blood transfusion reduces the level of HbS, thereby it is used to treat acute symptomatic anemia as well as treating and preventing many SCD complications [15]. To reduce the risk of stroke, especially in children with abnormal transcranial Doppler velocity, regular blood transfusion for at least one year is recommended to reduce HbS levels below 30% and to maintain Hb levels >9.0 g/dL. Assessment of iron overload is required to initiate iron chelators when indicated. Moreover, the ASH guideline suggests hydroxyurea therapy for children (ages 2–16 years) who live in low-middle-income areas (where regular blood transfusions and chelation therapies are not available or affordable). Hydroxyurea therapy decreases SCD-related complications (such as VOCs, ACS, hospitalization, and mortality rate) by increasing fetal hemoglobin (HbF) [15, 17].

SCD is a life-threatening disorder that is associated with acute and chronic complications interfering with daily activities and the quality of life of patients. In Sudan, SCD mainly affects children who continue to suffer from repetitive pain crises and frequent severe complications, and ultimately leads to early death. Therefore, specialized medical care focusing on prevention and regular assessment of disease management is urgently needed to reduce morbid events as well as the mortality rate. This study aimed to assess the management of sickle cell disease in pediatric patients at Gaafar Ibnauf specialized pediatric hospital in Sudan in light of international guidelines.

## 2. Methodology

### 2.1. Study Design and Setting

A descriptive, retrospective, hospital-based study was carried out in Gaafar Ibnauf Pediatric Hospital, Khartoum state, Sudan. Gaafar Ibnauf Hospital is the biggest referral specialized pediatric hospital in Sudan, receiving patients from all over the country. It encompasses nine units of different specialties in pediatrics (cardiology, neurology, gastroenterology, respiratory, hematology, and nursery).

### 2.2. Study Population

Study populations consisted of pediatric patients with SCD attending Gaafar Ibnauf Hospital and outpatient's clinic. All patients' files during the period (1 April 2018–1 July 2018) were manually screened.

#### 2.2.1. Inclusion Criteria

Medical files of pediatric patients with SCD attending Gaafar Ibnauf Hospital from April to July 2018 were included in this study.

#### 2.2.2. Exclusion Criteria

Incomplete patients records were excluded from the study.

### 2.3. Sampling Technique and Sample Size

A total coverage sampling technique was used in the current study. Two hundred and seven medical files of pediatric patients with SCD were selected based on the inclusion and exclusion criteria.

### 2.4. Data Collection Tool

The data were collected using a data collection sheet constructed by the researchers based on the study objectives. The data collection sheet consists of three parts. The first part covered the patients demographic data. The second part listed the common complications of SCD, and the third part covered diagnostic tests, management of the disease, and therapeutic monitoring throughout the scheduled follow-up visits.

### 2.5. Statistical Analysis

The data was analyzed using Statistical Package for Social Science Software (SPSS, version 20) and Microsoft Excel. For numerical data, the mean and standard deviation were calculated. For categorical data, descriptive statistics such as frequency and percentage were used to summarize the results. The Chi-square test was used to describe the association between variables. *P*value <0.05 was considered statistically significant.

### 2.6. Ethical Considerations

The ethical approval was obtained from the Research Board at the University of Khartoum, Faculty of Pharmacy (Research Ethics Committee, No. 59-5-3–2018). In addition, ethical clearance has been obtained from the Research Department, the Ministry of Health, Sudan. Permission to perform the study was obtained from the general director of the hospital. To ensure confidentiality of the patients' information, coded data collection sheets were used.

## 3. Results

### 3.1. Sociodemographic Characteristics of Patients

The study included 207 patients, with a mean age of 7.5 ± 3.1 years. More than half of patients (53.1%) were females, with a 1.1 : 1 female:male ratio and low socioeconomic status (53.6%). Only 4.3% had health insurance. Two-thirds of patients (66.2%) had no family history of sickle cell disease (Table 1).

### 3.2. Distribution of Sickle Cell Disease among Different Tribes in Sudan

Messeryia (11.1%) and Selehab (8.2%) tribes had the highest rate of SCD among the Sudanese tribes (Table 2).

### 3.3. Diagnostic Laboratory Tests for Sickle Cell Disease and Routine Laboratory Monitoring for Patients with Sickle Cell Disease

The major laboratory test that had been carried out for SCD diagnosis in the participants was hemoglobin electrophoresis (96.1%). All patients had been routinely monitored for hematological problems using a complete blood count test. 37.7% and 7.7% of patients had been monitored for liver and renal function, respectively. Transcranial Doppler ultrasonography (TCD) or pulmonary function tests were not routinely requested for the patients at each follow-up visit (Table 3).

### 3.4. Complications of Sickle Cell Disease among Participants

**Figure 1:**
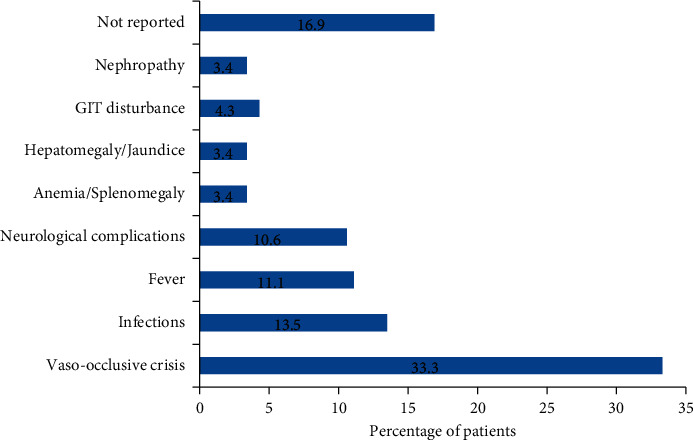
Complications of sickle cell disease among participants.

One-third of patients (33.3%) experienced vaso-occlusive crisis/pain, including 1% of patients having acute chest syndrome (ACS). 13.5% of patients had developed infections, with 9.6% infected with pneumococcal and meningococcal bacteria (respiratory tract infections, meningitis, and sepsis), whereas 3.9% had malaria infection. 4.3% of patients had experienced GIT symptoms. 11.1% and 10.6% of patients had experienced fever and neurological complications (such as hemorrhagic or ischemic stroke and headache). Anemia (hemolytic crises) had been encountered in 3.4% of patients, and 1.4% of the anemic patients had splenomegaly. Hepatomegaly/jaundice has been observed in 3.4% of patients (Figure 1).

### 3.5. Scheduled Follow-Up Visits in Patients with SCD

**Figure 2:**
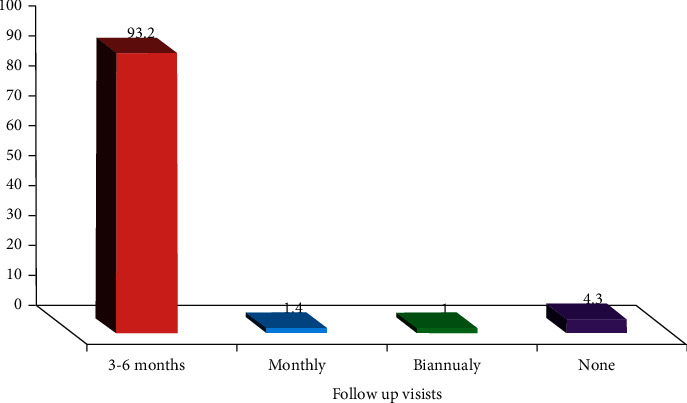
Distribution of patients according to the follow up visits.

The majority of patients (93.2%) had been scheduled for follow-up visits every 3–6 months (Figure 2).

### 3.6. Management of Patients with Sickle Cell Disease

This study showed that folic acid had been prescribed for all patients (100%), and 73.9% of patients had been treated with hydroxyurea therapy. The starting dose of hydroxyurea was 10 mg/kg/day and then the dose was escalated to 15 m/kg/day up to 35 mg/kg/day. The majority of patients received 15 mg/kg/day. Only 15% of patients received prophylactic penicillin with the majority of them (87.1%) receiving amoxicillin. More than half of patients (58.9%) were unvaccinated, and 41.1% of patients had been immunized with pneumococcal vaccine. All children younger than 2 years received 3 doses of the 13-valent pneumococcal conjugate vaccine (PCV13). Children 2–5 years old were administered 1–2 doses of PCV13 if they were unvaccinated or had received incomplete doses of PCV13. After completion of the PCV13 vaccine series, children aged ≥2 years received 1 dose of the 23-valent pneumococcal polysaccharide vaccine (PPSV23), with a booster dose 5 years later. More than two-thirds of patients (69.6%) were subjected to blood transfusions, with 13.9% (*n* = 20) of them undergoing chronic blood transfusions monthly for 3 years (Table 4).

### 3.7. Association between SCD Complications and Treatment Modalities

The use of hydroxyurea for the management of SCD was significantly associated with a low risk of fever (*P* *=* *0.045*). A significant reduction of vaso-occlusive crisis/pain (*P*=0.04) has been observed after blood transfusion (Table 5).

## 4. Discussion

Sickle cell disease (SCD) is an inherited blood disorder associated with acute and chronic complications and early death [18]. In Africa, thousands of children are born with SCD, and 90% of them die before the age of 5 years [3]. To date, there are no established programs in Africa for screening and clinical interventions for SCD management. Screening, pneumococcal prophylaxis, affordable treatment options, and caregivers' education effectively reduce child mortality due to SCD [19]. Providing optimal and comprehensive care to individuals with SCD can be challenging. Thus, implementing guidelines that provide evidence-based recommendations for the management of this life-threatening condition is of crucial importance and helps healthcare professionals to improve their practice [15]. This study aimed to assess the management protocol used for SCD in Gaafar Ibnauf Hospital in Sudan.

In this study, the participants were young children with a mean age of 7.5 ± 3.1 years. Most patients were females with low socioeconomic status. The results showed that 11.1% of the cases were from the Messeryia tribe in western Sudan. As reported in the literature, the prevalence of SCD in Sudan ranges from 2 to 30.4%, with a higher prevalence among tribes in western Sudan [6, 7, 20]. This could be due to migration from West African countries where the high prevalence of the disease is well documented [21].

In routine laboratory monitoring, all patients have been routinely monitored for complete blood counts, and unfortunately, transcranial Doppler ultrasonography (TCD) is not a routine practice at each follow-up visit. According to published guidelines, annual screening of stroke using transcranial Doppler ultrasonography is recommended for all children with sickle cell disease [15]. However, in Gaafar Ibnauf Hospital, TCD is only requested when neurological symptoms appear, and this practice could be due to the low socioeconomic status of the patients, unavailability, and unaffordability of the test.

Vaso-occlusive crises (VOCs), infections, fever, and neurological complications are the most serious complications that are reported during routine visits. Pneumococcal and meningococcal infections are the most commonly encountered complications in pediatric SCD. Hemorrhagic or ischemic stroke and headache are the most frequently encountered neurological complications in the current study. This finding is consistent with a previous study in Africa which demonstrated stroke as a common neurological complication of SCD [22]. In Africa, some complications such as silent brain infarcts, peripheral neuropathies, neurocognitive deficits, encephalopathy, or moyamoya disease are often underestimated because of the unavailability and unaffordability of diagnostic tests such as neuroimaging, transcranial Doppler ultrasonography, electroencephalogram (EEG), and neuropsychological evaluation [22]. Acute chest syndrome is a life-threatening complication, but it is not common in this study.

Anemia (hemolytic crises; Hb < 10 mg/dl) is one of the least complicated conditions in this study. This shows the effectiveness of the implemented treatment protocol in reducing the risk of anemia. The low incidence of hemolytic crises among the study participants reflects the adherence of physicians to the hospital treatment protocol regarding folic acid prescription for all patients and routine monitoring of hematological parameters. In this study, malaria infection and splenomegaly have been encountered in a few patients. According to the literature, splenomegaly is attributed to recurrent infections with *Plasmodium* species and is frequently reported in children with SCD (HbSS) in malaria-endemic countries [23]. These contradictory results seemed to be attributed to the early development of autosplenectomy in most children attending the hospital. Repeated attacks of VOCs cause autosplenectomy, rendering our patients more vulnerable to systemic infections. Autosplenectomy occurs because caregivers do not seek medical help at the earliest sign of the disease and most children start medications, especially hydroxyurea, at a later age (more than 9 months) after the onset of complications. This practice among patients could be related to a lack of good health education, low socioeconomic status, and health insurance coverage [24]. Moreover, most patients are from rural areas in western Sudan where there is a lack of health facilities. The low rate of malaria infection could be attributed to less exposure of patients to malaria infection by using preventive and control measures of malaria such as mosquito nets and repellents. Moreover, inaccessibility of screening tests and underreporting of complications may lead to underestimation of patients with splenomegaly and malaria.

In this study, most patients were unvaccinated and did not receive prophylactic penicillin. The high incidence of infections observed among participants in the current study may be explained by the failure in implementing an effective immunization protocol and the physician's nonadherence to guidelines, regarding antibiotic prophylaxis for children with SCD. Initiation of penicillin prophylaxis up to the age of 5 years, in addition to vaccination, is recommended to reduce the risk of developing serious bacterial infections [14, 15, 25]. Low prescription rates of prophylactic penicillin may be attributed to fear from the emergence of penicillin resistance, noncompliance of physicians to treatment guidelines, and nonadherence to medications in SCD patients [15, 26, 27]. Oral amoxicillin was the most frequently used prophylactic penicillin among the participants. Children with SCD in this study received only pneumococcal vaccine which was given according to clinical practice guidelines of pneumococcal immunization. Children younger than 2 years should receive 4 doses of the 13-valent pneumococcal conjugate vaccine (PCV13). Before starting the 23-valent pneumococcal polysaccharide vaccine (PPSV23), children 2–5 years old (unvaccinated or having received incomplete doses of PCV13) should be vaccinated with 1–2 doses of PCV13. After completion of the PCV13 vaccine series, children aged 2–18 years should receive 1 dose of PPSV23, with a booster dose 5 years later [28]. As indicated in SCD treatment protocols, immunizations should include all children's routine vaccines with the addition of the flu vaccine, pneumococcal vaccine, and meningococcal vaccine [14].

In this study, hydroxyurea was prescribed to most participants, reflecting the physician's adherence to SCD treatment guidelines regarding this medication. The starting dose of hydroxyurea was 10 mg/kg/day and escalated to a maximum tolerated dose (maximum dose is 35 mg/kg/day). The majority of patients received hydroxyurea at a dose of 15 mg/kg/day. Although oral L-glutamine was approved by the FDA in 2017 to reduce the acute complications of SCD in adult and pediatric patients older than 5 years [14,29], it has not yet been used or even registered in Sudan.

In this study, more than two-thirds of patients received blood transfusions, with 13.9% of them undergoing chronic blood transfusions every month for 3 years to prevent primary or secondary stroke. Chronic transfusion is used when unremitting reduction of HbS (less than 30%) is required for stroke prevention [15]. The majority of the patients in this study received episodic transfusions (periodic transfusions) for acute chest syndrome, acute anemia, hepatic sequestration, progressive intrahepatic cholestasis, priapism, and sepsis. Simple blood transfusion was frequently used in comparison with exchange blood transfusion.

In this study, few patients received analgesics for the management of painful crises. The SCD treatment guidelines strongly recommend immediate initiation of nonopioid and opioid analgesics for the management of pain associated with VOC, based on the level of patient-reported pain [15]. It appeared that most patients had mild pain, and the main barrier of the frequent prescription of NSADs is the side effects of these medications.

The scheduled follow-up visits are necessary for effective disease control. In this study, most patients had been scheduled for planned follow-up visits every 3–6 months. It has been observed from the medical history of patients that VOC episodes and hospitalizations usually occur before the three-month check-up period. Therefore, a three-month check-up period is rather long for monitoring the therapeutic outcomes of children with SCD. As an observation, some patients are unable to adhere to their follow-up visit, and this could be due to a lack of awareness of patients or their caregivers about the benefit of follow-up visits. In addition, financial issues may be a reason for nonadherence to follow-up visits since most patients are of low socioeconomic status and without health insurance.

This study revealed that hydroxyurea and blood transfusion for the management of SCD significantly reduced fever (*P: 0.0*4) and VOCs (*P: 0.04*), respectively. However, patients taking hydroxyurea appeared to be healthier with less frequent VOCs and other complications than those not taking hydroxyurea. As reported in the literature, both hydroxyurea and blood transfusion are used to reduce SCD-related complications such as recurrent vaso-occlusive crisis and fever [29]. Insignificant effects of hydroxyurea in reducing VOCs could be attributed to the use of relatively low doses of hydroxyurea (15 m/kg/day) in most patients or patients' nonadherence to hydroxyurea therapy. Hydroxyurea requires a monitoring protocol to ensure the highest benefits and safety of the therapy. Low doses of hydroxyurea have minimal effect in fetal hemoglobin production that mitigates tendencies for red blood cell sickling and VOC [15, 29]. A previous study demonstrated that hydroxyurea at a dose of approximately 30 mg/kg/day significantly reduces sickle cell-related adverse events (such as VOC) when compared to hydroxyurea at a dose of 20 mg/kg/day [30].

## 5. Limitations

The study is a single-center study with a relatively small sample size that may affect the generalizability of the results. In addition, some medical files had been excluded because of poor documentation.

## 6. Conclusion

The prevalence of SCD in Sudan is higher in the Messeryia tribe, originating from western Sudan. The treatment protocol at Gaafar Ibnauf Hospital-Sudan includes hydroxyurea, analgesics, folic acid, and blood transfusions. This treatment protocol, involving preventive and symptomatic therapy, is consistent with the international implemented protocols for the management of SCD in children. However, immunization and prophylactic penicillin approaches are deficient. The use of hydroxyurea and blood transfusion for children with SCD significantly reduces fever and vaso-occlusive crisis, respectively. Unfortunately, recently approved drugs, such as L-glutamine, have not yet been used in hospitals.

## 7. Recommendations

- Ongoing communication between healthcare providers and patients or caregivers on the use of hydroxyurea will enable informed joint decision-making and empower patients to initiate hydroxyurea therapy.
- Institution of comprehensive SCD centers focusing on life-long SCD management. Physicians, clinical pharmacists, and other healthcare workers involved in the care of SCD patients should be well trained and acquainted with current knowledge and standard practices in the treatment of SCD to improve treatment outcomes.
- Patients should be counseled on the need for adherence to scheduled vaccinations and ensure that all patients have received all vaccines that have been recommended by SCD treatment protocols.
- Communicate with health policymakers to provide free of charge medicines (hydroxyurea, folic acid, penicillin, and pneumococcal vaccine-23) and offer low-cost and high-quality laboratory tests for patients with SCD to increase the adherence to SCD treatment protocol.
- Intensify educational programs to enhance awareness of children and caregivers about the nature of the disease, possible complications that require immediate medical attention, the importance of antibiotic prophylaxis and vaccination to prevent life-threatening pneumococcal infections.

## Figures and Tables

**Table 1:** Patient's socio-demographic characteristics (*N* = 207).

Demographic Data	Mean	Std. deviation (%)
Age	7.5	3.1
Gender	Frequency	Percent
Male	97	46.9
Female	110	53.1
Socioeconomic status		
Low	111	53.6
Moderate	80	38.6
Unknown	16	7.7
Health insurance		
Yes	9	4.3
No	198	95.7
Family history of sickle cell disease		
Yes	64	30.9
No	137	66.2
Unknown	6	2.9

**Table 2:** Rate of sickle cell disease in the Sudanese tribes.

Sudanese tribes	Frequency	Percent
Messeryia	23	11.1
Selehab	17	8.2
Barno	14	6.8
Fallata	13	6.2
Bargo	13	6.2
Four	7	3.4
Rezegat	6	2.9
Rashyda	6	2.9
Hosa	6	2.9
Noba	5	2.4
Jammoeia	5	2.4
Zagawa	4	1.9
Benihalba	4	1.9
Bedireia	4	1.9
Taayisha	3	1.4
Omtenger	3	1.4
Rofaien	2	1
Masalti	2	1
Kanania	2	1
Hawazma	2	1
Gazami	2	1
Deedab	2	1
Dago	2	1
Berti	2	1
Baggara	2	1
Gaaline	2	1
Nemawia	1	0.5
Edasha	1	0.5
Danjo	1	0.5
Notrecorded	51	24.6

**Table 3:** Diagnostic laboratory tests and routine laboratory monitoring for patients with sickle cell disease.

Test	Frequency	Percent
Diagnostic laboratory tests		
Sickling test	4	1.9
Solubility test	3	1.5
Haemoglobin electrophoresis	199	96.1
HPLC	0	0.0
Isoelectric focusing test	0	0.0
Not reported	1	0.5
Routine laboratory tests		
CBC with Reticulocyte Count	207	100.0
Liver Function Test (LFT)	78	37.7
Renal Function Test (RFT)	16	7.7
EKG and Echocardiogram	7	3.4
Abdominal Ultrasound	4	1.9
Ophthalmology Test	2	1.0
Pulmonary Function Test	0	0.0
Transcranial Doppler Ultrasonography	0	0.0

**Table 4:** Management of patients with sickle cell disease (*N* = 207).

Management	Frequency	Percent
Folic acid	207	100.0
Hydroxyurea	153	73.9
Supplements	95	45.9
Antibiotics for infections	15	7.2
Analgesics	10	4.8
Prophylactic penicillins (*N* = 31)	31	15.0
Amoxicillin	27	87.1
Penicillin VK	4	12.9
None	176	85.0
Vaccination		
Pneumococcal vaccine	85	41.1
None	122	58.9
Blood transfusion		
Yes	144	69.6
No	63	30.4

**Table 5:** Association between complications of SCD and the treatment modalities.

Complications	Hydroxyurea (*N* = 153)		*Pvalue*
	Yes	No	
Fever	13 (8.5%)	140 (91.5%)	0.045^*∗*^
Vas-occlusive crisis	54 (35.3%)	99 (64.7%)	0.30
Blood transfusion (*N* = 145)			
Vas-occlusive crisis	42 (29%)	103 (71%)	0.04^*∗*^

^*∗*^ *P* ≤ 0.05

## Data Availability

The data supporting this research article are available from the corresponding author on reasonable request.
